# Effects of feet warming using bed socks on sleep quality and thermoregulatory responses in a cool environment

**DOI:** 10.1186/s40101-018-0172-z

**Published:** 2018-04-24

**Authors:** Yelin Ko, Joo-Young Lee

**Affiliations:** 1 College of Human Ecology, Seoul National University, Seoul, South Korea; 2 Research Institute of Human Ecology, Seoul National University, Seoul, South Korea; 3 COM:FORT Laboratory, College of Human Ecology, Seoul National University, 1 Gwanak-ro, Gwanak-gu, Seoul, 151-742 South Korea

**Keywords:** Feet warming, Sleep quality, Sleep-onset latency, Bed socks, Body temperature, Foot temperature, Distal-proximal skin temperature gradient

## Abstract

**Background:**

As a way of helping to sleep in winter, methods of warming the feet through footbaths or heating pads before bedtime are tried. In particular, bed socks are popular during winter sleeping in Korea, but scientific evidence about the physiological effects of bed socks on sleep quality is rarely reported. The purpose of this study was to evaluate the effect of feet warming using bed socks on sleep quality and thermoregulatory responses during sleep in a cool environment.

**Methods:**

Six young males (22.7 ± 2.0 years in age, 175.6 ± 3.5 cm in height, and 73.1 ± 8.5 kg in body weight) participated in two experimental conditions (with and without feet warming) in a random order. The following variables on sleep quality using a wrist actigraphy were measured during a 7-h sleep at an air temperature of 23 °C with 50% RH: sleep-onset latency, sleep efficiency, total sleep time, number of awakenings, wake after sleep onset, average awakening length, movement index, and fragmentation index. Heart rate and rectal and skin temperatures were monitored during the 7-h sleep. Questionnaire on sleep quality was obtained after awakening in the morning.

**Results:**

The results showed that sleep-onset latency was on average 7.5 min shorter, total sleep time was 32 min longer, the number of awakenings was 7.5 times smaller, and sleep efficiency was 7.6% higher for those wearing feet-warming bed socks during a 7-h sleep than control (no bed socks) (all *P* < 0.05). Also, their foot temperature was maintained on average 1.3 °C higher and the value in the distal-proximal skin temperature gradient was higher for those wearing feet warming bed socks when compared to the control condition (*P* < 0.05). However, there were no significant differences in heart rate, rectal and mean skin temperature, or in the questionnaire-based subjective evaluations between the two conditions.

**Conclusions:**

Feet warming using bed socks during sleep in a cool environment had positive effects on sleep quality by shortened sleep onset, lengthened sleep time, and lessened awakenings during sleep but had no significant influence on core body temperature. These results imply that sleep quality could be improved by manipulation of the foot temperature throughout sleeping.

## Background

Good sleep helps maintain a variety of bodily functions, such as enhancing the immune system, recuperation, and improving cognitive ability [1]. Since modern people are prone to a high degree of daily stress and to unhealthy lifestyles, getting recharged from a restful sleep is particularly relevant. In accordance with this, previous studies emphasizing the importance of sleeping in a comfortable environment to have a better sleep have proposed an optimal sleeping room temperature range of 25~27 °C [2, 3] based on “comfort mean skin temperature.” Besides such environmental issues, wearing “sleeping bed socks” while sleeping in winter also has been gaining popularity in South Korea as a personal measure of having a more sound sleep [4]. The sleeping bed socks are only for sleeping and not for everyday life, which are a little looser and made of softer materials when compared to daily socks.

According to Knab and Engel’s study, a better sleep can be represented by less time taken to fall asleep (shortened sleep onset time), less frequency of awakening, longer sleep time, less movements during sleep, and feeling more refreshed upon awakening [5]. To improve such sleep quality indicators, substantial studies on “external” treatments for better sleep have been conducted. The external treatments primarily utilized can be classified into two groups: one is setting up favorable sleeping environments [6–8] and the other is giving certain stimuli to the human body prior to or during sleep to induce desirable physiological responses, such as passive body heating to increase local skin temperature.

Based on the synchronization of sleep-wake and circadian body temperature rhythm, numerous observational studies have reported that local skin warming for the purpose of manipulating body temperature was effective for inducing good sleep [9–11]. There have been robust debates on the most effective skin location for passive body heating, but most correlational studies tended to investigate effects of warming distal body sites such as the feet and hands, which function as heat exchangers in humans due to their greater surface area ratio per unit body mass and particular cutaneous circulation. A warm footbath before sleeping is a widely utilized method for feet warming in many studies and has been shown to be beneficial for improving sleep quality by reducing the length of time taken to pass from wakefulness to sleep [10, 12–14]. Its effectiveness on accelerating sleep initiation was reported to be based on the functional link between sleep-onset latency and heat loss through distal skin regions before sleep, which can indirectly be measured by the distal and the proximal skin temperature gradient (distal-proximal skin temperature gradient; DPG) [15, 16].

Although the relationship between manipulating feet cutaneous blood circulation and good sleep has been well documented, there has been, as of yet, little evidence for the effectiveness of bed socks as a means of feet warming. Of the aforementioned studies, only the study of Raymann and colleagues used neutral and heated bed socks to warm feet before bedtime and for 90 min after the light had been turned off [13]. Locally manipulating foot temperature with bed socks is a noteworthy treatment in that it is not confined to a “before-sleep treatment” like using warm footbaths, which might be the reason for the previous studies’ primary focus on the effect of local warming on accelerating “sleep-onset” latency. Thus, it is advantageous to investigate the effectiveness of feet warming using bed socks through the whole sleep period. Also, since wearing bed socks during sleep is becoming more and more popular in South Korea, as already mentioned, its effectiveness needs to be proven. Such small change in the wearing habit might reduce indoor energy consumption as well by reducing heating during sleep in winter. That is, feet warming with bed socks during sleep at an air temperature 2~3 °C lower than the optimal temperature of bedroom reported may be able to contribute to saving indoor energy in cold winter.

For these reasons, the present study aimed to investigate the effects of feet warming using bed socks on sleep quality and the thermoregulatory responses during sleep in a cool environment of 23 °C. We hypothesized that feet warming during sleep would improve sleep quality in terms of sleep-onset latency and the frequency of awakening but would have no significant effect on core body temperature.

## Methods

### Subjects

Six Korean young male subjects participated in this study (mean ± SD 22.7 ± 2.0 years in age, 175.6 ± 3.5 cm in height, 73.1 ± 8.5 kg in body weight, and 23.6 ± 2.2 in body mass index). Volunteers having any symptoms of sleep disturbance, sleep disorder, or unusual (irregular) sleep patterns were excluded. We recruited subjects only who had a regular sleep schedule (6~8 h sleep per night) and daily activity routine through pre-screening. The subjects were also instructed to keep their daily activity routines before each experiment was conducted. Also, the subjects were required to abstain from strenuous exercise and alcohol 24 h before arriving, and to refrain from eating food including stimulating or caffeinated drinks 3 h before arriving, to minimize any effect of those factors on sleep quality. All the experimental procedures were described in detail to each subject who then signed to an informed consent prior to experiments. The experimental protocol was approved by the Institutional Review Board of Seoul National University (IRB No. 1801/001-004).

### Experimental clothing and bedding

**Fig. 1:**
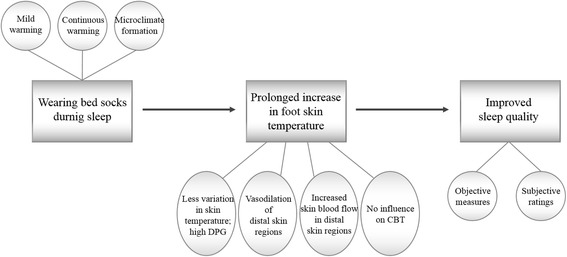
The conceptual and methodological framework of the present study

The conceptual and methodological frameworks are illustrated in Fig. 1. Subjects were identically dressed in long-sleeved T-shirts, long sweat pants, and undershorts (670 g in total, 0.66 clo in estimated clothing insulation). The top cuff circumference, cuff length, foot length, fabric thickness, mass per area, and air permeability of bed socks were 16.5 cm, 12.0 cm, 20.0 cm, 2.4 mm, 0.0316 g/cm^2^, and 20.1 cm^3^/cm^2^/s at 125 hPa, respectively. The fabric composition of the bed socks was polyester 98.6% and polyurethane 1.4%. The socks were worn in one of the two conditions and not reused. When moving into a climatic chamber from a preparation room to sleep, subjects wore in-house slippers and were required to take them off before stepping into the chamber. Each subject was assigned to single bedding, which includes a sponge mattress (polyester 100%, 5 cm thickness) and its sheets (cotton 100%, 110 cm × 185 cm), a blanket (cover: cotton 100%, 160 cm × 200 cm, padding: polyester 100%, 3.5 cm thickness), and a pillow (cover: cotton 100%, 40 cm × 60 cm, padding: polyester 100%, 15 cm thickness). In both control and feet warming condition, subjects wore the identical bedding during sleeping.

### Experimental conditions and procedures

Experiments in this study consisted of two conditions: one condition in which subjects slept wearing no socks (CON) and the other condition in which subjects slept wearing bed socks to warm their feet (Feet warming condition). At least 2 days before each experiment day and between the two experiment conditions, subjects were instructed to keep their own regular sleep patterns. Subjects were required to visit the laboratory twice in total to participate in each 7-h sleep experiment. Each visit was separated by an interval of 6 days on average (at least 2 days) to help the subjects to regain their regular sleep schedules before revisiting the laboratory for the second experimental condition. The order of the conditions was randomized to avoid the effect of familiarization. In both conditions, each subject slept in a climatic chamber at an air temperature (*T*_a_) of 23.0 ± 0.1 °C with 55.0 ± 0.7% relative humidity (RH) from 00:00 AM to 07:00 AM.

**Fig. 2:**
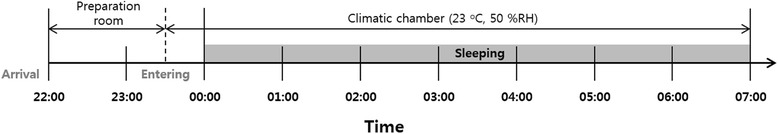
Experimental procedure according to time

Subjects were required to arrive at the laboratory at least 2 h before each scheduled test and after changing into experimental clothing, rest sufficiently. A group of three subjects were tested together, and the subjects became closer for the 2~3 h before starting the experiments. In the Feet warming condition, bed socks were worn approximately 1 h before going to bed to sufficiently manipulate foot skin temperature prior to the start of measurements. At 11:25 PM, subjects were asked to leave the preparation room. Before moving into the bedroom in the climatic chamber at 11:30 PM, subjects were asked to use the bathroom if needed and not to bring any kind of personal electrical devices into the bedroom. In case of emergency during experiments, subjects were encouraged to go out of the bed room immediately and let the experimenters know. After entering the bedroom, subjects lay down on a mattress which was placed on the floor and covered themselves with a blanket assigned to each person, resting their heads on a pillow. Closing eyes was discouraged until 00:00 AM to prevent subjects from falling asleep before measurements start. Each subject was instructed to start sleeping in a proper position at 00:00 AM with lights off, wearing earplugs provided to exclude influence of unnecessary noise on sleep. At 07:00 AM, lights were turned on and subjects were woken up. After being directed to the preparation room and completing a sleep quality questionnaire about the previous night, all the sensors and experimental clothing were removed from subjects (Fig. 2).

### Measurements and calculations

Over a period of 7 h, rectal temperature (*T*_re_) was measured every 30 s by a thermistor probe inserted 16 cm beyond the anal sphincter. Skin temperatures on the ten sites (the forehead, chest, abdomen, forearm, hand, thigh, calf, foot, ankle, and sole) were measured every 30 s by thermistor probes. All the skin temperature probes were attached on the left side of the body except for the forehead, which was measured in the center. Ankle temperature was measured on the back. *T*_re_ and skin temperatures were recorded automatically using a data logger (LT-8A; Gram Corporation, Japan). Mean skin temperature (${\overline{T}}_{sk}$) was calculated using a Hardy and Dubois’ equation [17]. Distal-proximal skin temperature gradient (DPG) was calculated by subtracting the proximal temperature (mean of the chest, thigh, abdomen, and forehead skin temperature) from the distal temperature (mean of the hand and foot skin temperature) [16]. Negative value in DPG hence indicates that proximal skin temperatures were higher than distal and vice versa for positive value in DPG. Heart rate was monitored every 1 s (RC3 GPS, Polar Electro, Finland), and the data were sorted out at the interval of 30 s. Total sweat rate (TSR) was estimated using a change in total body mass before and after the experiment (ID2, Mettler-Toledo, Germany; resolution of 1 g). The wrist-worn Actigraphy (wGT3X-BT, Actigraph, FL, USA) monitor, a tri-axial accelerometer, was worn on the non-dominant side of each subject to obtain objective sleep variable data. The primary sleep variables measured were sleep-onset latency (SOL—the amount of time in minutes taken to be scored as “asleep” by an algorithm), total counts (TC—the sum of total counts of actigraphy during the whole period of sleep; activity was “counted” by wrist-worn actigraphy when it caused the acceleration signal to exceed the threshold, and the number of activity “counts” would be calculated at the end of the measurement period; larger total counts indicate that there were more activities counted [18]), sleep efficiency (SE—the number of “asleep” minutes divided by the number of minutes of the whole sleep period), total sleep time (TST—the number of minutes in total scored as “asleep” during the sleep period), wake after sleep onset (WASO—the number of minutes in total when the subject was “awake” after sleep onset), number of awakenings (NOA—the number of awakenings per sleeping period scored by the algorithm; or frequency of awakening), average awakening length (AAL: the average length of “awaken” period in minutes). There were three indexes with regard to sleep fragmentation which represent the degree of being non-relaxed during the sleep period: movement index (MI) and fragmentation index (FI), and sleep fragmentation index (SFI). Greater values in MI, FI, and SFI indicate that sleep was more disrupted.

### Sleep quality questionnaire

Subjective sleep quality was measured using a developed sleep quality questionnaire. Each questionnaire filled in by each subject was completed without being interrupted. The ten-question questionnaire consisted of two parts: Part 1 (questions 1 to 7) asked the degree of fragmentation and depth of the previous sleep, and part 2 (questions 8 to 10) obtained a subjective evaluation of the bedroom environment in terms of temperature, humidity, and thermal comfort.

Seven questions asking about fragmentation and depth of sleep were developed from Verran and Snyder-Halpern (VSH) Sleep Scale factors [19]. Five out of seven questions used a 5-point scale, even though the VSH Sleep Scale followed a visual analog format, to help subjects respond with ease [1. “Estimate of the amount of movement during sleep”: 0 Tossed all night, 1 Tossed frequently, 2 Neutral, 3 Hardly tossed, 4 Did not toss at all; 2. “Estimate of depth”: 0 Slept lightly, 1 Slept somewhat lightly, 2 Neutral, 3 Slept somewhat deeply, 4 Slept deeply; 3. “Estimate of how rested you are upon awakening”: 0 Awoke exhausted, 1 Awoke somewhat exhausted, 2 Neutral, 3 Awoke somewhat refreshed, 4 Awoke refreshed; 4. “Spontaneity with which you awoke in the morning”: 0 Awoke abruptly, 1 Awoke somewhat abruptly, 2 Neutral, 3 Awoke somewhat spontaneously, 4 Awoke spontaneously; and 5. “Estimate of sleep along dimensions of satisfaction, quality, and disturbance”: 0 Bad night, 1 Somewhat bad night, 2 Neutral, 3 Somewhat good night, 4 Good night. Such modification between categorical scales and visual analog scales was validated by Lee et al. [20]. Similar to the VSH Sleep Scale, the total scores of five questions were calculated. The higher the total score was, the better the quality of sleep. Two out of seven questions asked subjects to directly write down “6. Estimate of number of awakenings during the sleep period” and “7. Estimate of amount of time from settling down to sleep until falling asleep.”

One question inquiring about temperature of the bedroom was provided using a 9-point scale [8. How hot was the room you slept in: − 4 Very cold, − 3 Cold, − 2 Cool, − 1 Slightly cool, 0 Neutral, 1 Slightly warm, 2 Warm, 3 Hot, 4 Very hot]. Two questions on humidity and thermal comfort of the bedroom were given with a 7-point scale [9. How humid was the room you slept in: − 3 Very dry, − 2 Dry, − 1 A little dry, 0 Neutral, 1 A little wet, 2 Wet, 3 Very wet; 10. How thermally comfortable was the room you slept in: − 3 Very uncomfortable − 2 uncomfortable − 1 A little uncomfortable, 0 Neutral, 1 A little comfortable, 2 Comfortable, 3 Very comfortable].

### Data analyses

Sleep variable analyses with Actigraphy data were performed with Actilife 6 using the Sadeh algorithm [21]. All temperature and heart rate data were averaged into 30 min for analytical and graphical purpose. The normality of the data was tested using the Shapiro-Wilks test. To examine differences in physiological and questionnaire responses between the two conditions, the paired *t* test and the Wilcoxon signed-rank test were used, with parametric data and with non-parametric data, respectively. All the statistical analyses were undertaken using SPSS Statistics 24.0. Significance level was set at 0.05.

## Results

### Sleep variables

**Fig. 3:**
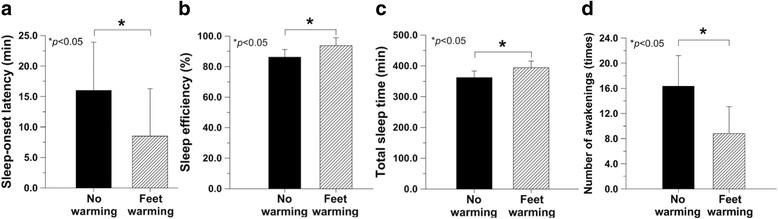
Sleep-onset latency (**a**), sleep efficiency (**b**), total sleep time (**c**), and number of awakenings (**d**) (*N* = 6)

Sleep-onset latency was significantly shorter in Feet warming condition than in CON (*P* = 0.018), showing 7.5 ± 5.3 min of difference (Fig. 3a). Sleep efficiency was significantly improved by 7.6 ± 7.1%, which were 93.8 ± 5.1% in Feet warming and 86.2 ± 5.1% in CON (*P* = 0.047) (Fig. 3b). Total sleep time was also significantly 32.0 ± 29.9 min higher when wearing bed socks (Feet warming 394.0 ± 21.5 min) than with no socks (CON 362.0 ± 21.4 min) during sleep (*P* = 0.047) (Fig. 3c). Subjects showed significantly fewer number of awakenings during 7 h of sleep when wearing bed socks than in CON (*P* = 0.041). Number of awakenings when feet were warmed by bed socks was 8.8 ± 4.3 times, while the number was 16.3 ± 4.9 times when no bed socks were worn (Fig. 3d). Total counts for Feet warming condition seemed to be higher than that in the CON condition, but the difference was not significant (*P* = 0.056). Wake after sleep onset, average awakening length, movement index, fragmentation index, and sleep fragmentation index did not significantly differ (Table 1).

**Table 1:** Summary of physiological responses and variables from actigraphy

Variables	No warming (CON)	Feet warming	*P* value
Sleep-onset latency [min]	16.0 ± 7.9	8.5 ± 7.8	0.018
Sleep efficiency [%]	86.2 ± 5.1	93.8 ± 5.1	0.047
Total sleep time [min]	362.0 ± 21.4	394.0 ± 21.6	0.047
Number of awakenings [times]	16.3 ± 4.9	8.8 ± 4.3	0.041
Total counts [times]	14,314 ± 3303	7926 ± 5497	0.056
Wake after sleep onset [min]	42.0 ± 20.4	17.5 ± 14.5	0.078
Average awakening length [min]	2.5 ± 1.0	1.8 ± 1.1	0.345
Movement index (MI)^a^ [%]	14.52 ± 2.55	9.09 ± 5.20	0.079
Fragmentation index (FI)^b^ [%]	11.07 ± 7.72	5.36 ± 9.41	0.328
Sleep fragmentation index (SFI)^c^ [%]	25.59 ± 10.09	14.45 ± 9.36	0.134
Decreases in rectal temperature for 7-h sleep [°C]	0.63 ± 0.39	0.54 ± 0.31	0.427
Averaged mean skin temperature for 7-h sleep [°C]	35.1 ± 0.2	35.2 ± 0.3	0.660
Averaged distal-proximal skin temperature gradient (DPG) for 7-h sleep [°C]	− 0.78 ± 0.56	− 0.19 ± 0.50	0.043
Insensible body mass loss for 7-h sleep [g h^−1^]	42.4 ± 16.7	52.6 ± 10.3	0.280
Increases in ankle skin temperature at 2.5 h [°C]	3.7 ± 2.6	4.8 ± 2.9	0.547
Averaged ankle skin temperature for 7-h sleep [°C]	33.6 ± 0.6	34.3 ± 0.4	0.003
Averaged foot skin temperature for 7-h sleep [°C]	34.2 ± 0.4	35.0 ± 0.2	0.012
Averaged hand skin temperature for 7-h sleep [°C]	34.4 ± 1.0	34.8 ± 0.7	0.287
Averaged heart rate [bpm]	61 ± 7	63 ± 6	0.245

All data were expressed as mean ± SD

^a^*MI* the percentage of periods with positive value in the *y*-axis counts during the sleep period

^b^*FI* the percentage of 1 min periods of sleep versus the whole periods of sleep

^c^*SFI* the sum of MI and FI (*N* = 6 except for the foot temperature [*n* = 5])

### Rectal temperature (*T*_re_), mean skin temperature (${\overline{T}}_{\mathrm{sk}}$), DPG, and TSR

**Fig. 4:**
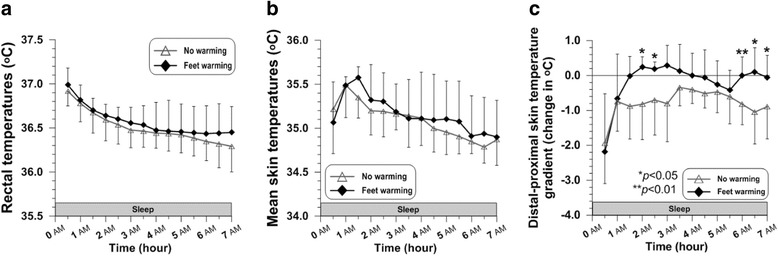
Time courses of rectal temperature (**a**), mean skin temperature (**b**), and distal-proximal skin temperature gradient (**c**) during sleep (*N* = 6)

Rectal temperature in both conditions (CON and Feet warming) continuously declined during sleep, with no significant difference (Fig. 4a). Differences in this decrease in rectal temperature during sleep was insignificant, 0.63 ± 0.39 °C for CON and 0.54 ± 0.31 °C for Feet warming (Table 1). There was also no significant difference in mean skin temperature, which increased at the beginning of sleep and gradually decreased after (Fig. 4b). Distal-proximal skin temperature gradient (DPG) was significantly higher when wearing bed socks than with no socks at 2 h (*P* = 0.044), at 2.5 h (*P* = 0.028), at 6 h (*P* = 0.002), at 6.5 h (*P* = 0.026), and at 7 h (*P* = 0.028) (Fig. 4c). The time taken to elevate the DPG value above 0 °C was 2 h in Feet warming, whereas it never reached 0 °C during the whole period of sleep in no warming. Averaged DPG in Feet warming (− 0.19 ± 0.50 °C) was also significantly greater than that in CON (− 0.78 ± 0.56 °C) (*P* = 0.043) (Table 1). Total sweat rate (TSR) showed no statistical difference between the two conditions. During 7 h of sleep, insensible perspiration occurred as much as 42.4 ± 16.7 g h^−1^ in CON while TSR in Feet warming was 52.6 ± 10.3 g h^−1^ (Table 1).

### Skin temperatures on the extremities: the ankle, foot, and hand

**Fig. 5:**
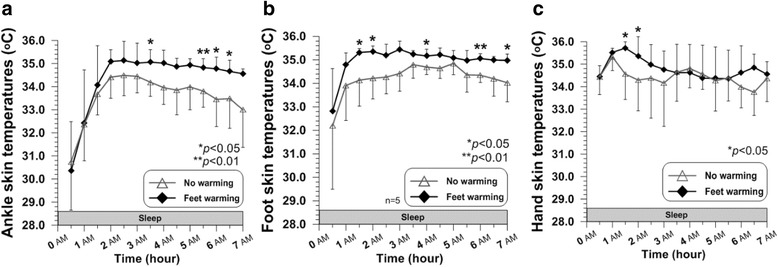
Time courses of ankle skin temperature (**a**), foot skin temperature (**b**), and hand skin temperature (**c**) during sleep (*N* = 6 except for the foot temperature [*n* = 5])

Ankle skin temperature increased until at 2.5 h by 3.7 ± 2.6 °C in CON and by 4.8 ± 2.9 °C in Feet warming without significant difference (Table 1). Increased skin temperature on the ankle in Feet warming seemed to be better maintained until before waking up, showing significantly higher temperature than that in CON at 5.5 h (*P* = 0.004), at 6 h (*P* = 0.013), and at 6.5 h (*P* = 0.037) (Fig. 5a). Averaged ankle skin temperature for 7 h of sleep in Feet warming (34.3 ± 0.4 °C) was significantly higher than that in CON (33.6 ± 0.6 °C) (*P* = 0.003) (Table 1). Subjects showed significantly greater foot skin temperature in Feet warming than in the other condition at 1.5 h (*P* = 0.043), at 2 h (*P* = 0.032), at 4 h (*P* = 0.036), at 6 h (*P* = 0.0031), and at 7 h (*P* = 0.03) (Fig. 5b). Wearing bed socks during sleep induced significantly higher average foot skin temperature for 7 h in Feet warming (35.0 ± 0.2 °C), compared to that in CON (34.2 ± 0.4 °C) (*P* = 0.012) (Table 1). Hand skin temperatures in Feet warming was significantly greater than that in CON only at the relatively early stage of sleep, which were at 1.5 h (*P* = 0.046) and at 2 h (*P* = 0.022) (Fig. 5c). Average hand skin temperature during the whole time of sleep in Feet warming was 34.8 ± 0.7 °C and that in CON was 34.4 ± 1.0 °C, but the difference was not significant (Table 1).

### Heart rate

**Fig. 6:**
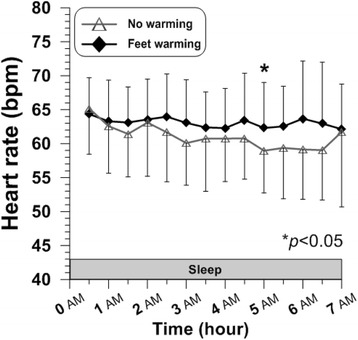
Time courses of heart rate during sleep (*N* = 6)

Heart rate was within normal ranges in both conditions throughout the sleep period, without any statistical significant differences except for at 5 h (*P* = 0.024) (Fig. 6). Averaged heart rate for 7 h also did not show significant difference between the two conditions (Feet warming 63 ± 6 bpm, CON 61 ± 7 bpm) (Table 1).

### Sleep quality questionnaire responses

The total score of sleep quality in terms of the amount of movement, sleep depth, degree of being rested and spontaneity when waking up, and the comprehensive evaluation of the previous sleep was higher in Feet warming than that in CON with no significant difference. Subjects also reported that the bedroom they slept in was warmer, less humid, and more thermally comfortable, but the differences were not significant (Table 2).

**Table 2:** Responses of ten questions from sleep quality questionnaire

Questions	No warming (CON)	Feet warming	*P* value
1. Estimate of the amount of movement during sleep	1.17 ± 0.90	1.33 ± 0.94	N.S.
2. Estimate of depth	1.67 ± 0.75	1.67 ± 1.11	N.S.
3. Estimate of how rested you are upon awakening	2.00 ± 1.00	2.50 ± 0.96	N.S.
4. Spontaneity with which you awaked in morning	0.50 ± 0.50	1.17 ± 0.90	N.S.
5. Estimate of sleep along dimensions of satisfaction, quality, and disturbance	1.83 ± 1.07	2.00 ± 0.82	N.S.
Total scores of sleep quality (sum of #1~5)	7.17 ± 2.54	8.67 ± 3.73	N.S.
1. Estimate of number of awakenings during the sleep period [times]	1.67 ± 0.47	2.00 ± 1.53	N.S.
2. Estimate of amount of time from settling down to sleep until falling asleep [min]	47.17 ± 61.46	37.50 ± 31.06	N.S.
3. How hot was the room you slept in	− 0.17 ± 1.67	0.33 ± 1.11	N.S.
4. How humid was the room you slept in	0.17 ± 0.69	0.00 ± 0.58	N.S.
5. How thermally comfortable was the room you slept in	1.17 ± 0.69	1.83 ± 0.37	N.S.

All data were expressed as mean ± SD (*N* = 6)

## Discussion

### Local body warming and sleep quality

It is well documented that skin temperature on the extremities increases prior to sleep and is associated with sleep propensity in terms of sleep onset and initiation of the first period of slow-wave sleep, which is often referred to as deep sleep stage [22–24]. Along with these observational findings, various studies have manipulated foot temperature through local warming, showing it to quicken sleep initiation. In the present study, manipulating skin temperature on feet by wearing bed socks caused significant increases in average foot and ankle skin temperature during sleep. When subtle local warming on feet was applied (Feet warming condition), sleep quality also appeared to improve not only with shortened sleep initiation but also with higher sleep efficiency, longer total sleep time, and fewer awakenings during a 7-h sleep. The presumable neurobiological mechanisms for its effectiveness on enhancing sleep propensity might be associated with hypothalamic activation [13]. Warm-sensitive neurons (WSNs) which account for 30% of the neurons in the preoptic area and anterior hypothalamus (POAH), the major thermoregulatory region of the brain, have been shown to respond to changes in skin temperatures and subsequently increase their firing rate [25–27]. According to the following findings, increased neuronal activity of WSNs can be interpreted as facilitating sleep: the firing rate of WSN increases at sleep onset, at the onset of slow wave sleep, and in slow wave sleep; it decreases prior to the onset of wakefulness and in wakefulness [28–30]. It is plausible therefore to assume that elevated foot skin temperature by local warming using bed socks might activate the thermosensitive neurons raising their discharge rate in brain areas in charge of sleep regulation [31] and have a beneficial impact on enhancing the quality of a 7-h sleep.

In some of the previous studies in which foot temperature was manipulated by a warm foot bath prior to sleep, however, feet warming was evaluated not to have remarkable effects on sleep quality [11, 13, 14]. The conflicting results in those previous studies and the current study can be attributed to the different methods of local feet warming. Although footbath has been a well adopted means of examining the correlation between warmed feet and sleep quality being reported to be effective in the other studies, it can only be applied before or at the beginning of sleep. Wearing bed socks, in contrast, is not only applied before sleep starts but also can be worn, thereafter making it possible for mild feet warming effectiveness to last throughout the whole sleep period, which deserves to be noted.

### Distal-proximal skin temperature gradient and sleep quality

Due to the difference in thermophysiological regulatory mechanisms between proximal and distal skin temperatures, circadian rhythms of proximal skin temperature and of distal show an inverse relationship. During awakening period, proximal skin temperature maintains higher than distal skin temperature. However, during sleep, distal skin temperatures increase and proximal skin temperatures decrease. Distal-proximal skin temperature gradient (DPG) hence increases and even reaches above 0 °C during sleep [32]. In the present study, averaged DPG in Feet warming condition was significantly higher and the sleep quality determined from the sleep indicators appeared to be higher as well, which is in accordance with the findings proposing it to be the best physiological indicator of sleep initiation [15, 16]. Higher DPG in Feet warming, around 0 °C in particular, indicates that there was little variance in skin temperatures and the body has changed its state into “the completely relaxed one-compartment body” due to the practical disappearance of the thermoregulatory shell [32].

Higher DPG driven by elevated foot temperature has been proposed as an indirect measure of increased distal skin blood flow occurring due to vasodilation of distal skin regions and hence larger heat loss via the extremities [33]. The functional relationship between distal vasodilation and sleep was shown in the finding of Kräuchi and colleagues [16]. In addition to being used as a selective indicator of increased “local” blood flow, it has been further suggested that even “whole” body skin blood flow might be evaluated using DPG as whole body circulation can be promoted by distal vessel dilation [10]. A conceivable deduction of the effect of increased skin blood flow driven by wearing bed socks on sleep quality in the present study is that its influence on limiting blood supply to other organs such as the brain [31]. Cerebral blood flow hence decreases presumably indicating that less activity occurs in the brain; this conforms to several early sleep investigations: Cerebral blood flow is reduced during the transition from wakefulness to sleep and less in the sleep stages compared to before-sleep. And especially during slow wave sleep stages, cerebral blood flow to all brain regions is at its minimum level [34–36].

### Core temperature and sleep quality

It is well known that the sleep-wake cycle is closely related to the circadian rhythm of core body temperature. During the daytime, the core temperature remains high and peaks between 4 PM and 8 PM, continuously decreasing thereafter at night which has been documented as a signal for the human body to prepare for sleep [37]. This decline is determined by heat exchange between the core and the shell and heat redistribution in the human body during sleep [23]. The largest decline rate in core temperature has long been investigated to be involved in sleep initiation and the onset of the main sleep period in numerous studies [38, 39]. Several studies, however, suggest this decline has less effect on sleep regulation than the changes in skin temperature. Kräuchi and colleagues found a weaker correlation between core temperature or rate of change and sleep propensity than between the distal proximal skin temperature (DPG) and sleep onset, which is an indirect measure of heat loss via the extremities [16] as mentioned earlier. The importance of increased heat loss on sleep is supported by evidence from other studies. When core body cooling is applied during daytime, both core temperature and especially distal skin temperatures decreased, followed by reduced sleepiness indicating that sleepiness is not always induced with reduction in core temperature but can be reduced by decreased distal skin temperature [40]. A core temperature decrease hence may rather be a consequence of heat loss, not a key cause of sleep initiation [41], while circadian and behavior-induced skin temperature variance associated with sleep may function as an input signal to the sleep control system [31].

In the present study, passive body heating on the feet using bed socks during a 7-h sleep did not have a significant effect on core temperature but significantly improved sleep quality. This is in accordance with the findings of previous studies in which sleep-onset latency was accelerated in young adults by a warm footbath (42 °C) for 30 min [9, 12] or with heated bed socks before sleep but no difference in core temperature [13]. The results obtained in regard to core temperature and sleep quality seem to be driven by the mildness of feet warming method that can raise skin temperature without stimulating thermoregulatory heat defense systems against the heat stress induced so that sleep propensity was not reduced by the treatment [23]. It also implies that a steeper decrease in core temperature is not necessarily required to enhance sleep quality when increased skin blood flow can be induced by local skin warming, supporting the idea as described above that the core temperature per se within normal ranges is less involved in sleep propensity.

Moreover, it is worth to note the microclimate created around the feet by wearing bed socks. The isolated microclimate may have acted to limit too much heat loss to a cool bedroom environment, which can occur due to prolonged increased skin blood flow. Although elevated skin blood flow seems to be beneficial to sleep quality, too robust heat dissipation can lead to a too steep decrease in core temperature, thereby causing hypothermia and an extremely inefficient energy metabolism. Effectively restricting heat transfer from the distal body sites to the environment with an isolated environment reduces heat flow as well as enables skin blood flow to increase [31]. So, it is supposable that the decline in core temperature for feet warming using bed socks may have been less triggered despite the distal skin blood flow increase, as transferred heat from the core to the periphery was limited to be discharged on the distal sites because of the microclimate. It is also conceivable to attribute insignificant difference between no warming and Feet warming in core temperature during sleep to the bedroom climate designed to be neither too cold nor too hot. In other words, the mild cool air temperature, only in a slightly lower level of the indoor temperature of bedroom in winter, chosen for this study may induce sufficiently comfortable sleep without wearing socks.

### Heart rate during sleep

It is generally accepted that heart rate decreases in normal human sleep compared to that in wakefulness. When “permitted” to sleep, heart rate abruptly decreases. Combined with the increase in skin temperatures at the initiation of sleep, Kräuchi and Wirz-Justice reported that rapid fall in heart rate might represent a measure of consequent shift in heat redistribution from the core to the periphery [41]. Lowered heart rate changes during sleep depending on different sleep stages [42], and it shows a rising phase just before awakening, according to the findings from the study of Degaute et al. [43]. The normal heart rate range in young healthy males during sleep reported in the several previous studies was approximately 50 to 70 bpm [42, 44, 45], which covers the heart rates observed in both conditions of the present study. In the current study, there was no remarkable difference in heart rate between the two conditions, while sleep quality seemed to be improved when feet were warmed. Feet warming using bed socks while sleeping does not necessarily seem to induce any significantly different responses in heart rate.

### Subjective evaluation and sleep quality

Although warming feet by wearing bed socks was expected to have beneficial effects on subjective ratings of sleep quality, no significant results were observed. In some of the previous studies, however, passive body heating of the feet was reported to have positive influences on evaluation of perceived sleep quality [12]. Insignificant differences in subjective ratings of sleep quality in the present study might be the result of the small number of subjects who participated, which may be insufficient to statistically analyze difference in subjective evaluation. In addition, the sleep environment designed in our study with only slightly lower air temperature than that in preferred bedroom climate in winter as mentioned earlier can be considered as sufficiently thermally comfortable. This mild climatic environment might have rendered subjects unable to subjectively differentiate the sleep qualities between wearing socks and no socks during sleep, making it less effective on enhancing subjective sleep propensity.

### Limitation and suggestion

The findings from the current study should be further investigated with a larger sample of subjects and also include the elderly and females. Pathological studies can also be conducted with subjects who have various types of sleeping disorders, to evaluate clinical effectiveness of feet warming using bed socks. In addition, similar tests in colder climatic environments are needed to examine the advantages of feet warming using bed socks worn during sleep as a home-applicable method to improve sleep quality. Further studies investigating the effectiveness of feet warming using bed socks while keeping regular sleep schedules of each subject which the present study did not reflect can also be conducted. Last but not least, the neurobiological mechanisms quoted in the present study are presumed to underlie the correlation of improved sleep quality and feet warming using bed socks, based on not actual experimental results but deduction. Therefore, further studies are necessary to draw more clear understanding of its underlying mechanism built on results of animal studies.

## Conclusions

The present study found that feet warming using bed socks during the whole 7-h sleep period improved sleep quality by accelerating sleep initiation and maintaining the relaxation of the body while sleeping, without disturbing the operations in the homeothermic core. An increase in distal skin temperature induced by wearing bed socks during sleep seems to be the mechanism at work in enhancing sleep propensity. The physiological mechanisms for its effectiveness might be due to modulating the firing properties of sleep-active neurons and reducing cerebral blood flow, which were triggered by reducing differences in skin temperature resulting in evenly distributed heat throughout the body. The findings of the present study are applicable to the daily bedroom environment, yet further investigation with more subjects as well as a broader range of subjects is needed.

## Abbreviations

AAL: Average awakening length
DPG: Distal-proximal skin temperature gradient
FI: Fragmentation index
MI: Movement index
NOA: Number of awakenings
POAH: Preoptic area and anterior hypothalamus
RH: Relative humidity
SE: Sleep efficiency
SFI: Sleep fragmentation index
SOL: Sleep-onset latency
T(_)sk: Mean skin temperature
*T*_a_: Air temperature
TC: Total counts
*T*_re_: Rectal temperature
TSR: Total sweat rate
TST: Total sleep time
VSH: Verran and Snyder-Halpern
WASO: Wake after sleep onset
WSNs: Warm-sensitive neurons
